# Barriers to hospital-based phase 2 cardiac rehabilitation among patients with coronary heart disease in China: a mixed-methods study

**DOI:** 10.1186/s12912-022-01115-6

**Published:** 2022-11-29

**Authors:** Xiaoqi Xie, Qiongshan Chen, Hui Liu

**Affiliations:** 1 Shantou University Medical College, Shantou, Guangdong Province China; 2 Department of Cardiology, The Second Affiliated Hospital of Shantou University Medical College, Shantou, China

**Keywords:** Nursing, Cardiac rehabilitation, Coronary heart disease, Mixed-methods study, Barrier, Covid-19

## Abstract

**Background:**

Coronary heart disease (CHD) has become a leading cause of morbidity and premature death worldwide. Cardiac rehabilitation (CR) was proved to have substantial benefits for patients with CHD. The CR was divided into three phases. Phase 2 is the important part of CR which involves hospital-based structured and closely monitored exercises and activities. However, CR utilization is low worldwide. The barriers to hospital-based phase 2 CR in China have not been well identified.

**Aims:**

To investigate barriers to hospital-based phase 2 cardiac rehabilitation among coronary heart disease patients in China and to explore the reasons.

**Methods:**

This study employed an explanatory sequential mixed-methods design. The study was conducted in a university hospital in China from July 2021 to December 2021. Quantitative data was collected through the Cardiac Rehabilitation Barrier Scale. Qualitative data was collected through unstructured face-to-face interviews. Data analysis included descriptive statistics and inductive qualitative content analysis.

**Results:**

One hundred and sixty patients completed the Cardiac Rehabilitation Barrier Scale and 17 patients participated in unstructured face-to-face interviews. The main barriers identified were distance (3.29 ± 1.565), transportation (2.99 ± 1.503), cost (2.76 ± 1.425), doing exercise at home (2.69 ± 1.509) and time constraints (2.48 ± 1.496). Six themes were identified; logistical factors, social support, misunderstanding of cardiac rehabilitation, program and health system-level factors, impression of CR team and psychological distress. The first four themes confirmed the quantitative results and provide a deeper explanation for the quantitative results. The last two themes were new information that emerged in the qualitative phase.

**Conclusion:**

This study provides a better understanding of the barriers to hospital-based phase 2 cardiac rehabilitation among coronary heart disease patients in the Chinese context during the Covid-19 pandemic. Innovative programs such as home-based CR, mobile health, and hybrid programs might be considered to overcome some of these barriers. In addition, psychosocial intervention should be included in these programs to mitigate some of the barriers associated with the impression of CR team and psychological distress.

## Introduction

Coronary heart disease (CHD) has increasingly become a leading cause of morbidity and premature death worldwide [1]. China, as a middle-income country, has an estimated 11 million patients with CHD, which accounts for the largest proportion of cardiovascular diseases (CVDs) nationwide [2]. The mortality of CHD was 126.9/100000 in urban areas and 135.88/100000 in rural areas in China in 2020 [2]. The incidence of CHD has been continuously increasing and will continue to increase in the next decade [2].

Cardiac rehabilitation (CR) is a comprehensive continuum of care for patients with CHD after initial treatment. Normal, the CR was divided into three phases. Phase 1 starts in the hospital which focusing on recover basic functional mobility. Phase 2 begins when patients discharge from hospital which involves structured and closely monitored exercises and activities. It is often lasted for at least 3–6 months after discharge. Phase 3 focus on keeping up exercises and maintain a healthy life style [3]. The benefits of CR are clear, including improving cardio-pulmonary function, promoting wellness, and improving quality of life [4, 5]. In addition, CR was proven to reduce cerebrovascular events, hospital readmission rates, and mortality rates [6].

Regardless of the well-described benefits of CR, CR utilization remains low around the world. Worldwide, CR overall referral rates were 43%, enrollment was 42%, and adherence was maintained for 70% of prescribed sessions [7]. In America, the data from the US Catheterization/PCI Registry of 1310 hospitals between 2009 and 2012 revealed that 59.2% of patients were referred to CR [8]. The USA most recently reports 24% of patients actually enrolled. After enrollment, 57% of these patients adhered to ≥25 CR sessions, and 27% completed the full 36 prescribed sessions [9]. 36–54% of primary PCI patients attended CR in Australia [10]. In Europe, self-reported CR utilization at up to 131 hospitals in 27 countries revealed 46% of patients were referred, with 69% of those reporting they attended at least half of prescribed sessions [11]. In China, although hospitals with CR centers have increased significantly in the past 5 years, the participation rate remains quite low, with approximately 5% enrollment [12, 13].

It is challenging to increase CR utilization. Effective intervention can only be proposed after adequate investigation of the barriers. CR barriers occur at multiple levels, including physicians, patients, and systems [14]. In low- and middle-income countries, the most commonly reported barrier was the lack of physician referral. Patient-related factors were affordability, particularly due to lack of insurance coverage, transportation difficulties, primarily driven by long distances to CR centers, an unwillingness to attend CR, and competing priorities on patients’ time. The most frequently reported systems factor was the lack of personnel and resources. In addition, self-efficacy, self-motivation, self-esteem, personality, depression, anxiety and social support were reported to be barriers to CR [15].

Some research has focused on ethnic minorities or women. Language was reported as the main barrier in treating ethnic minorities [16]. The language barrier was associated with a lack of understanding of verbal and written instructions, a lack of certified translators to facilitate communication between ethnic minority groups and the CR team, a lack of interaction with the CR team and a lack of communication about feelings and disease-related information. Age and comorbidities, a history of depression, transportation problems, family obligations, a lack of CR insurance, financial concerns, and a lack of social support from family and friends were the most common barriers identified by women [17]. Some qualitative studies indicated that key barriers were background knowledge, in-the-moment understanding, personal responsibility, social connectedness and perceived benefits [18].

China is still in the preliminary stage of developing CR. According to a recent global survey of CR programs, 216 hospital-based CR programs are available in China [19]. However, only a few studies have investigated the barriers to CR access. One qualitative study conducted in Nanjing reported that the main barrier was affordability [12]. Another quantitative study conducted in 11 hospitals in Shanghai identified that distance, lack of awareness, weather and transportation were the main barriers [13]. Affordability was not identified as a barrier, which conflicts with the finding of the qualitative study conducted in Nanjing. Another quantitative study aimed at cultural adaptation of the Cardiac Rehabilitation Barriers Scale (CRBS) in one hospital in Beijing, China, but the author did not report any data about the scores acquired [20].

The previous three studies in China were conducted in Beijing, Nanjing and Shanghai, which are culturally different from the city of Shantou. Shantou is the largest city in the Chaoshan region, with a population of 5.64 million. The regional dialect is Chaoshan, which is considered one of the most difficult Chinese dialects to learn and is reportedly spoken by more than 70% of the population in the Chaoshan region. A recent cross-sectional study in Shantou reported that linguistic barriers significantly impact health care delivery with perceived adverse impacts on the ability of the entire health care system to operate effectively [21].

In addition, a recent global cross-sectional study completed by 1062 CR programs in 70 countries indicated that the COVID-19 pandemic has impacted CR programs worldwide, including the cessation of services, a decrease in CR components delivered, a change in treatment mode delivery without much opportunity for planning and training, and psychosocial and economic impacts on health care providers [22]. Another study reported that, in the current pandemic era, an extra 7.3 million employees, including their families, have become unemployed [23]. The restrictions of the pandemic and the influence of restrictions on the economy might add new barriers to CR programs. Government restrictions in response to the COVID-19 pandemic have varied widely across different countries, and even in different areas of the same country. Therefore, it is necessary to explore the barriers to CR during the pandemic.

There are few studies focusing on CR barriers in China, and the number of CR programs is increasing, so barriers to access should be assessed in China. In addition, China is a large country with diverse cultures, which might be potential barriers to CR delivery. Moreover, the restrictions of the pandemic and the influence of restrictions on the economy and mental health might have added some new barriers to CR programs. Last, previous studies have been solely quantitative or qualitative. No previous research has explored CR barriers by using mixed -methods design in China.

Therefore, this study investigated the barriers to hospital-based phase 2 cardiac rehabilitation among patients with CHD in Chaoshan, China, during the COVID-19 pandemic by using mixed methods. It will provide a better and deeper understanding of barriers in the current Chaoshan region in China during the pandemic and provide evidence for the design of future intervention programs.

## Methods

### Study design

This study uses an explanatory sequential mixed-methods design. The study included a quantitative and a subsequent qualitative phase. Quantitative data were collected and analyzed first, and then qualitative data were collected to explore the quantitative results in an in-depth way [24]. The quantitative phase identified the patients who were recruited for the qualitative phase and guided question development [25]. This mixed method design enables researchers to use a qualitative lens to explore findings of quantitative results by exploring the participants’ view in greater detail and thus better describe missed data than either method alone [25].

### Setting and participants

#### Setting

The study was conducted in a university hospital located north of the city center (Shantou).

The hospital has approximately 1400 beds with a PCI center, two cardiology units, a coronary care unit (CCU) and a CR center. The CR center is the first to be certified as meeting national standards in the city of Shantou, and it provides structured and standard phase 1 to phase 3 CR programs. The annual program capacity for phase 2 CR is approximately 1800 patients. This capacity is sufficient for current needs, and no patients need to wait. The CR center is available during the weekdays. Phase 2 CR normally lasts for 3–6 months, including 36 sessions. A virtual program is not currently provided in the center.

During the research period, patients came to the center after making an appointment online first, and when they entered the hospital entrance, they needed to accept a primary COVID-19 screening, which included showing the “Yuekang Code” and “Itinerary Card”, and having their temperature taken. Individuals who have a fever or cough, whose Yuekang code is red or yellow, who have a recent history of living in medium- and high-risk areas within the past 21 days, or have a history of touching imported cold chain food will be guided to the fever clinic and directed to take a nucleic acid (PCR) test, routine blood test and chest X-rays (including CT scans if necessary). With negative PCR test results, patients were allowed to go to the outpatient clinic. The PCR test can be done in most of the public hospital and it usually need at least 4 hours waiting for the result. The result is valid within 72 hours.

#### CR protocols

The CR program is comprehensive in our hospital and follows the cardiac rehabilitation guideline [26] and the Expert Consensus of China for CR among patients with CHD [27]. When patients are hospitalized, phase 1 CR is initiated within 24 hours. The CR nurses who are educated and qualified for the CR program consult with the patients and perform a risk factor assessment, mobilization assessment and readiness for CR assessment (phase 1 CR consent forms are signed at this time). Then, based on the results of the assessment, the CR nurse will discuss with the doctor in charge and other CR team members (including the pharmacist, dietitian, psychological therapist or psychological counselor, and smoking cessation counselor) and make a phase 1 CR plan together for the patient. The day before discharge, patients are automatically referred to the outpatient phase 2 CR program through our health information system (HIS) and are invited to join our CR WeChat group for continuous management and care if they meet the phase 2 CR criteria. The CR nurse will introduce the phase 2 CR program to patients and ask about their willingness to participate. If they agree, they will be invited to sign a consent form for phase 2 CR. From 1 week to 1 month after discharge, patients who signed up to participate in phase 2 CR visit our CR outpatient clinic and are asked to perform a cardiopulmonary exercise test (CPET) or a 6-minute walking test and a series of assessments. After that, an exercise program is prescribed. Then, patients follow the schedule as prescribed to join monitored exercise sessions in our CR center as well as receive health education.

Patients who agreed to participate in the phase 2 CR program and signed the consent form during the CR education session before discharge were defined as enrollers. Patients who attended the first assessment and one hospital-based monitored CR session in phase 2 within 3 months of discharge were considered participants. Patients who finished the prescribed CR sessions within 6 months of discharge were considered to have completed CR.

#### Participants

For the quantitative session, a number of consecutive inpatients with CHD between July 2021 and December 2021 were recruited from the two cardiology units and CCU. The inclusion criteria were as follows: 1) age 18 or more; 2) no severe comorbidities (tumor malignancies, severe liver and kidney disease, severe lung disease); and 3) ability to speak and listen in Mandarin or the local language (Chaoshan language). The exclusion criteria included patients with psychiatric illness or cognitive decline. According to the general requirements of multivariate analysis, the sample size is expected to be 5–10 times the number of variables (items on the scale), and an additional 20% should be added to sample size to take into account the potential loss of participants, so our study needed at least 126 cases [28].

For the qualitative part, purposive sampling was used until achieving data saturation. At last, a subsample of 17 participants who completed the survey and refused to participate in the phase 2 CR participated in unstructured, in-depth, face-to-face interviews. In each session, patients were invited to sign the informed consent form. All methods were carried out in accordance with relevant guidelines and regulations in the Helsinki Declaration**.**

### Data collection

#### Quantitative data collection

The survey began with items regarding sociodemographic as well as clinical characteristics. Sociodemographic and clinical characteristics were collected through the health information system (HIS) and patients’ self-report. The Cardiac Rehabilitation Barriers Scale (CRBS) was used to investigate patient barriers to CR. The original English version of the CRBS consists of 4 domains and 21 items. The scale was translated into Mandarin, cross-culturally adapted and psychometrically validated, including 21 items in five domains that were confirmed to have acceptable validity and reliability [13]. Patients rated their level of agreement with each CRBS item on a 5-point Likert scale, with response options ranging from 1 (strongly disagree) to 5 (strongly agree). Higher scores indicated greater barriers to CR [21]. Averages of the CRBS subscales were calculated from the scores on each item included in the subscale. Face-to-face structured questionnaires were used during data collection. Each participant spent approximately 10 to 15 minutes answering the survey. Data collection for the quantitative phase took place from July 2021 to December 2021 by the authors on the day before each patient was discharged.

#### Qualitative data collection

A purposive sample was used to recruit participants for interviews to maximize the depth and richness of the data [25]. Participants who finished the quantitative survey and refused to participate in the phase 2 CR were contacted and invited to participate in the qualitative phase. Patients who agreed and signed the informed consent form were arranged to be interviewed. The interviews happened while the patients were still inpatients.

An in-depth, face-to-face unstructured interview was used as the main data collection approach. The interview was conducted in a private room in the CCU. Interviews began with friendly conversation. The interview was recorded by using a digital recorder. The interview began with an open question, “Can you tell me your thoughts or feelings about being invited to participate in the CR program?” After this open question, subsequent questions were asked to understand the barriers to their decision-making. Some probing questions were asked, such as “can you tell me what’s keeping you from coming to cardiac rehabilitation?” or “can you tell me a little bit more about what are you worried about?” The interviews were flexible enough to generate richer information. Patients were allowed to add any information they deemed to fit the questions.

The interview lasted for approximately 30 minutes to 45 minutes. During the interview, field notes were taken to record the nonverbal behavior and activities of participants. When no more new information was emerging, data saturation was considered to be achieved, and then data collection was ended.

### Data analysis

#### Quantitative data analysis

The survey data were analyzed by using SPSS Statistics Version 25 (SPSS Inc.). The normality test for the scores of the whole scale and subscales were performed first. A descriptive statistic, including frequencies, percentage, mean and standard deviation was performed to examination of participant characteristics, as well as the scores of CRBS.

##### Qualitative data analysis

Qualitative data were analyzed by using inductive qualitative content analysis [29]. The data analysis was concurrent with the data collection process. After each interview, the researcher transcribed the data within 1 week. The recording was listened to again and again to gain familiarity with the scope of the content of each data source and build a contextualized and holistic understanding of the participants. During the transcription process, the tone of the voice, silences, and pauses of the participant were noted. The transcripts were cross-checked and labeled by the first and second authors independently. After the transcript was completed, line-by-line coding began and these codes were grouped into categories [30]. The categories were compared for similarities and differences and then grouped into more abstract levels (themes).

#### Integration of the quantitative and qualitative data

Data integration was conducted in the design, methods, and interpretation stages [24]. The study had an explanatory sequential design that integrates quantitative data first and then qualitative data [19]. For the methods, the sample in the qualitative phase was selected from the sample in the quantitative phase. In the interpretation part, the quantitative and qualitative results were compared, integrated and then jointly displayed.

### Validity and reliability/rigor

The CRBS (Chinese version) used in the quantitative phase was tested to have acceptable validity and reliability [13]. Member checking was used to enhance the credibility. All participants in this study were invited to review the transcript to ensure that the transcript reflected their real thinking. In addition, peer review was conducted by inviting two colleagues who are familiar with this area to review the transcript and the findings. Researchers discussed with colleagues to ensure that we did not bring our own assumptions to the findings. An audit trail was conducted to improve dependability. During data analysis, we did not participate to ensure that the data analysis was not colored by our previous experience or idea.

## Results

### Quantitative findings

A total of 160 participants completed the survey. Among the 160 patients, 126 (78.7%) patients participated in phase 1 CR, which is a relatively higher rate; however, only 39 patients were assigned to participate in phase 2 CR, and ultimately, only 9 patients completed the prescribed CR program. Fifty-three (33.1%) patients were living in the city or town, while the other 69% of patients were living in rural areas, suburban areas or other cities. Other sociodemographic and clinical characteristics of the participants are shown in Table 1.

**Table 1:** Characteristic of participants

	Total (*n* = 160)
**Sociodemographic**	
Age in years, n (%)	
45 or younger	19(11.9)
46–60	52(32.5)
61–80	85(53.1)
80 or older	4(2.5)
Gender, n (%)	
Male	126(78.8)
Female	34(21.3)
Marital status (%married)	148(92.5)
Nationality (% Han)	159(99.4)
Residence (% city or town)	53(33.1)
Education, n (%)	
Junior high school and below	82(51.2)
Technical secondary school/senior high school	69(43.1)
College degree	9(5.6)
Work status (% working)	67(41.9)
Monthly income, n (%)	
<3000RMB	78(48.8)
3000-8000RMB	74(46.3)
>8000RMB	8(5.0)
Healthcare insurance coverage, n (%)	
Government or insurance	129(80.6)
Out-of-pocket	31(19.4)
**Clinical characteristics**	
CABG (% yes)	1(0.6)
PCI (% yes)	112(70)
Heart failure (% yes)	16(10)
Hypertension (% yes)	86(53.8)
valvular heart disease (% yes)	5(3.1)
Diabetes (% yes)	42(26.3)
hyperlipidemia (% yes)	66(41.3)
Tobacco use(% yes)	94(58.8)
Family history of CVD (% yes)	10(6.3)
Regular exercise (% ≥3 times/wk. for ≥30 min) (% yes)	38(23.8)
BMI	26.17 ± 19.069
Participant in the phase 1 CR (%)	126(78.7)

*BMI* body mass index, *PCI* percutaneous coronary intervention, *CABG* coronary artery bypass grafting

The distance from the CR facility was rated as the most significant barrier to enrollment (3.29 ± 1.565), followed by transportation problems (2.99 ± 1.503) and CR costs (2.76 ± 1.425). Other significant barriers (average value approximately 2/5) included “I already exercise at home, or in my community” (2.69 ± 1.509), time constraints (2.48 ± 1.496) and work responsibilities (2.43 ± 1.666) (Table 4).

### Qualitative results

A total of 17 participants completed the interview. Their sociodemographic characteristics are presented in Table 2. Six themes were identified after analyzing the content of the transcriptions, namely, logistic factors, social support, misunderstanding of CR, program and health system-level factors, impression of health care providers and hospital surroundings and psychological distress (Table 3).

**Table 2:** Sociodemographic of interview participants

Sociodemographic	Total ***n*** = 17
Age in years, n (%)	
45–60	9(52.9%)
61–80	8(47.1%)
Gender, n (%)	
male	10(58.8%)
Female	7(41.2%)
Marital status (%married)	11(64.7%)
Nationality (% Han)	17(100%)
Residence (% city or town)	8(45.5%)
Education, n (%)	
Junior high school and below	8(47.1%)
Technical secondary school/senior high school	9(52.9%)
Work status	
Working n(%)	8(47.1%)
Retired or have no work n(%)	9(52.9%)
Monthly income, n (%)	
<3000RMB	7(41.2%)
Over 3000RMB	10(58.8%)
Healthcare insurance coverage, n (%)	
Government or insurance	5(29.4%)
Out-of-pocket	12(70.6%)
Regular exercise (% ≥3 times/wk. for ≥30 min) (% yes)	7(41.2%)
Undergone PCI (Yes)	9(52.9%)
Participant in the phase 1 rehabilitation (Yes)	12(70.6%)

**Table 3:** Themes and categories of the findings

Themes	Categories
**Logistical Factors**	Distance
	Inconvenient traffic
	Insufficient economic support
	Parking difficulty
**Social support**	Lack of family support
	Caregiver role conflict
	Work Conflict
**Misunderstanding of CR**	Believing that daily activities can replace CR
	Doubt the effectiveness of CR
**Program and health system-level factors**	Limited CR centers and inflexible time
	Covid-19 test
**Psychological distress and personality**	Pessimism
	Anger and Hostility
	Escaping
**Impression on cardiac team**	Believe doctors rather than nurses

#### Theme 1: logistical factors

##### Distance

Patients from nearby cities or rural areas claimed they could not participate due to the distance involved. When asked how far would be acceptable, most of them said that more than 40 minutes of driving distance would be unacceptable.

P4: *“It is too far. I live in Chenghai. It takes me more than 40 minutes to drive here.”*

P6: *“I cannot take part in it. My home is too far away from here. It takes me approximately 40 or 45 minutes even if I drive on the expressway.”*

##### Inconvenient traffic

Some patients came to the hospital by public bus. However, public buses are limited in some areas, and sometimes there is no direct route to the hospital. Therefore, some patients needed to transfer many times, which wasted a great deal of time on the road. Meanwhile, some patients said that they lived in rural areas where there is no public transportation. This was a large barrier for them.

P3: *“There is no direct bus. I have to transfer three times.”*

P7: *“It takes me half an hour to get here by bus and I have to spend more than one hour to wait for the bus, it is so inconvenient.”*

P9: *“There is no public transportation in my living area. Therefore, I need to call the taxi every time. It is truly an inconvenience.”*

##### Parking difficulty

Some participants thought there was limited access to parking at the hospital. Sometimes they needed to find parking space outside the hospital and then walk to the hospital. It was not convenient.

P1, P12 and P15: *“Hospital parking drives me crazy.”*

P5: *“The parking service is terrible, you know, it is always full.”*

##### Insufficient economic support

CR is cost-intensive and is not covered by medical insurance. For patients with poor economic conditions, costs were identified as a major barrier.

P7: *“It’s too expensive. I really cannot understand why this (CR) is not included in my medical insurance?”*

P8, P11: *“I have no income. I [already] borrowed money from relatives to pay for the expensive cardiac surgery. I do not have extra money to do the rehabilitation.”*

P16: *“I heard that this (cardiac rehabilitation) will cost at least thousands of dollars a month, and I can’t afford it.”*

#### Theme 2: social support

##### Lack of family support

Family support played an important role in the CR program. Most elderly patients came to the hospital with accompanying family members. However, their family members could not always provide support for them.

P3: *“It takes my son two hours to drive me here. It’s too far. My son needs to work, and he can’t take me to here every time.”*

P6: *“My son has to go to work, so he can’t pick me up every time.”*

##### Caregiver role conflict

P2: *“My grandchild is only 4 years old. His father and mother are working in another city. I need to take care of my grandchild”.*

P4: *“I know rehabilitation will benefit me, but I have so much housework to do: go to the market every day, cook three meals for my daughter’s whole family… I do not think I have spare time to take part in CR.”*

P8: *“I am in charge of everything at home. I have to pick up my two grandchildren, buy food, cook, wash clothes and mop the floor. There is no time for CR.”*

##### Work conflict

Some adults have to return to work after cardiac events. It is difficult to participate in CR due to the conflict between work hours and rehabilitation time.

P9: *“I have to work after discharge. You know, I cannot stop. I have to pay the mortgage every month. It is impossible for me to do rehabilitation two times each week on a workday. You know, the CR center does not open on the weekend.”*

P10: *“I operate a water and electricity decoration company. Now it is the end of the year, and many families need to decorate their house. I have a lot of work to do now, and I have been rushing to make sure everyone can move into their newly decorated house before the Spring Festival. I don’t have enough time to arrange space in my schedule for rehabilitation.”*

#### Theme 3: misunderstanding of rehabilitation

##### Daily activities can replace CR

Although CR has been promoted in China for more than 10 years, the public awareness of rehabilitation is inadequate. Patients do not have a correct understanding of CR and think that daily activities and exercise outside the hospital can replace CR.

P1: *“I go to the gym near my house every day.”*

P5: *“I have a treadmill at home, and I insist on running every day.”*

P9: *“For exercise, I can exercise at home after I leave the hospital.”*

P11: *“There are a lot of fitness machines downstairs in my community. I can do that in the community.”*

##### Doubting the effects of CR

P9:*“ I just want to treat the disease, there is no need to do rehabilitation.”*

P10: *“After surgery, the most important thing is to reduce exercise to help the body recover.”*

P15: *“This is a big surgery for me (PCI), I probably need half to one year to recover. Surgery has exhausted my qi (yang qi), I need to stay at home quietly and drink traditional Chinese medicine to help restore the yang qi.”*

#### Theme 4: program and health system-level factors

##### Limited CR centers and inflexible time

There are only a few CR centers, most of which are in third-class A general hospitals, and these hospitals are always located in the center of the town.

P1 and P9: *“The center opens from 8 am to 5:30 pm from Monday to Friday and it is closed on weekends. You know, for me, I only have time on weekends. Therefore, that might be the biggest obstacle for me.”*

P10: *“If the center (cardiac rehabilitation center) was like those convenience stores which opened everywhere, it might be easier for me to get access.”*

##### COVID-19 test

P7 and P14: *“When I go to the hospital, I need to show the negative result for a COVID-19 test; I feel burned out from that.”*

P7: *“I heard that the result (COVID-19 test) was only valid for 72 hours. It means I need to do the test every time when I come. It is too much trouble.”*

#### Theme 5 impression of the cardiac team

For some participants, their impression of the health care providers, especially the person who referred them to the CR center, impacted their decision-making process. Patients believe that doctors’ advice is more believable and valuable than nurses’ advice.

##### Believing doctors rather than nurses

P12:*“It is so strange that a nurse comes to my bed and says I need the CR program. Who is this nurse? Why didn’t my doctor recommend this to me?”*

##### Impression of surroundings

P12: *“When the nurse brought me to the CR center, I saw so many people (both men and women) running on the treadmill; it is so strange that men and women exercise in the same room.”*

##### Impression of health care providers

P17: *“You know, the nurse informed me that taking part in the program will help me lose my weight. I know, I am too fat. However, you know, the nurse is fatter than me actually. I do not believe that she can help me lose weight.”*

#### Theme 6 psychological distress and personality

##### Pessimism

Some participants explained their cardiac disease in a very negative way and thought that rehabilitation was useless for them. Some people expressed their conditions with a sense of hopelessness.

P3: *“It is unfair, you know, I did a lot of good things in my life. I always supported others and helped others. I thought I would get good fortune in my life. However, it is not....... I don’t want to think about these things (rehabilitation). Let it go......”*

P16: *“My mother-in-law is 83 years old; she does nothing every day. Her son (my husband) died 26 years ago. I have no choice but to take care of her. She is healthy without any disease. So why did I get sick? Why did I get the heart attack? It is unfair. Is it not?”*(Tears in her eyes).

##### Escaping

Some patients expressed their wishes to leave the hospital as soon as possible after discharge.

P6: *“Please do not ask me anything about my heart. I do not believe I have heart disease.”*

P11: *“The hospital is a dirty place with a lot of unlucky things. It was a cemetery many years ago. It’s horrible, I don’t want to stay here.”*

P15: *“Do not ask me to come to the hospital every week. I want to stay at home.”*

##### Anger and hostility

P6: *“I am an experienced coach. Do you want to teach me how to do exercise?”* (with a contemptuous smile).

P9: *“Exercise in the hospital? Are you kidding? I think your hospital just wants to get more money from me.”*

P12: *“The surgery cost me a lot. You want to get more money from me?”*

### Mixed-methods findings

Some findings from the qualitative research confirmed the findings from quantitative research and helped explain the quantitative results in more detail.

For the first domain (logistical factors), distance and transportation problems were the most important barriers with higher scores. In the qualitative phase, these barriers were confirmed, and participants mentioned that more than 40 min of driving distance might be a cutoff point. In addition, participants who live in rural areas without public transportation seemed less likely to take part in the program.

The cost of CR, in the quantitative part, focused on the cost of transportation and gas. However, in the qualitative part, it focused on the program cost, which was not covered by outpatient medical insurance in China. Participants needed to pay the expensive assessment fee (such as cardiopulmonary exercise testing) and the 36 session guided exercises fee by themselves. Some participants thought this would be a large burden for them.

Severe weather was not evaluated as an important barrier in either the quantitative or qualitative results. The lack of parking space at the hospital was a barrier that emerged in the qualitative phase.

In terms of the second domain (CR need), “I already exercise at home, or in my community” was identified as a barrier. This was also confirmed by the qualitative results. Participants thought they could go to the gym, or some of them had a treadmill at home. They thought rehabilitation was the same as normal physical activity.

For the third subscale (time conflicts), the qualitative results confirmed that caregiver role conflict and work conflict were the main barriers.

Therefore, most of the results from quantitative research were confirmed by the results from qualitative research. However, during the qualitative phase, some new themes emerged. These include the impact of the COVID-19 restrictions, impressions of health care providers and hospital surroundings, psychological distress and personality. This supplements the quantitative findings. The results of both the quantitative and qualitative phases are jointly displayed in Table 4.

**Table 4:** Integration of quantitative and qualitative results

Subscales	Items	Scores	Qualitative Themes	Categories	Merging/Integrating results
Domain 1 logistical factors	1…of distance	3.29 ± 1.565	Theme1 logistics factors	Distance	Confirmed by quantitative and qualitative results Details: more than 40 minutes driving distance seems too far for participants
	2…of cost	2.76 ± 1.425		Insufficient economic support	Confirmed by quantitative and qualitative results However, the detail of cost is different. In quantitative result, the cost focus on gas, parking, but in qualitative result, the cost refers to grogram cost. The program cost was not covered by outpatient medical insurance.
	3…of transportation problems	2.99 ± 1.503		Inconvenient traffic	Confirmed by quantitative and qualitative results. Detail: living in rural areas without public transportation
	8…severe weather	1.73 ± 0.888			In qualitative result, no people mentioned weather as a barrier
				Parking difficulty	Detail: lack of parking space in hospital and it costs time to find the parking space
Domain 2 CR need	5…I did not know about cardiac rehab	1.61 ± 0.825	Theme2 Misunderstanding of CR		Not a big barrier. In qualitative part, all patients claimed they were referred and they have heard cardiac rehabilitation
	6…I do not need cardiac rehab	1.82 ± 1.069		Doubt the effect of cardiac rehabilitation	Confirmed by quantitative and qualitative results. In qualitative part, some participants said they just want to treat the disease, there is no need to do rehabilitation
	7…I already exercise at home, or in my community	2.69 ± 1.509		Daily activities can replace rehabilitation	Confirmed by quantitative and qualitative results. Detail: participant think they can go to the gym or some of them have treadmill at home. They think rehabilitation is the same as physical activities.
	17… many people with heart problems do not go, and they are fine	1.66 ± 0.785			
	18… I can manage my heart problem on my own	1.68 ± 0.879			
	21…I prefer to take care of my health alone, not in a group	1.70 ± 0.896			
Domain 3 Time conflicts	4…of family responsibilities	2.17 ± 1.299	Theme3 Low social support	Lack of family support Caregiver role conflict	In qualitative part, most participants claimed that take care of their grandchildren or family members is a barrier.
	10…travel	1.74 ± 1.019			The center opens on daytime and just open from Monday to Friday, it is conflict with the working time
	11…of time constraints	2.48 ± 1.496			
	12…of work responsibilities	2.43 ± 1.666		Work Conflict	In qualitative part, some younger patients claimed that they need return to work and there is no spare time to do the rehabilitation in the center
Domain4 Program and health system-level factors	16…my doctor did not feel it was necessary	1.54 ± 0.743	Theme4 Program and health system-level factors		
	19… I think I was referred, but the rehab program did not contact me	1.64 ± 0.842			
	20…it took too long to get referred and into the program	1.65 ± 0.818		Limited cardiac rehabilitation center	No CR center near their home
				Covid-19 test	New information emerged in qualitative phase. It is a trouble to show the negative result every time when enter the hospital
Domain5 Comorbidities/ Functional status	13…I do not have the energy	1.99 ± 1.171			No information emerged in this domain in the qualitative phase
	14…other health problems prevent me from going	1.75 ± 0.938			
	9…I find exercise tiring or painful	2.00 ± 1.208			
	15…I am too old	1.86 ± 1.141			
			Theme5 Impression on healthcare team		New information emerged in qualitative phase Referred by physicians or cardiologists is better than referred by nurses
			Theme 6 Psychological distress	Escaping	New information emerged in qualitative phase
				Anger and Hostility	New information emerged in qualitative phase
				Pessimism	New information emerged in qualitative phase

## Discussion

The results of this mixed-method study indicated multilevel barriers for patients with CHD to take part in the hospital center-based phase 2 CR programs. The distance from the CR facility was rated as the most significant barrier to enrollment, followed by transportation problems and CR costs. Other significant barriers included “I already exercise at home, or in my community”, time constraints and work responsibilities.

Distance has been identified as the first major barrier, which is in accordance with most previous studies [13, 31, 32]. In the qualitative interview, participants reported that normally more than 40 minutes of driving distance would be a cutoff point for them. They were unlikely to attend the CR if they needed to spend more than 40 minutes on the road. A previous study reported that patients are significantly less likely to enroll in CR where they must drive 60 minutes or more to the closest program [33]. Therefore, we suggest that health care providers take geography into consideration when referring patients to CR.

Transportation was identified as the second CR barrier, which has also been widely suggested in previous studies [13, 34]. Specific reasons found in the qualitative part are that patients living in remote areas often face a lack of public transportation. Even if there are public buses, they need to wait for a long time for them to arrive. Therefore, they think it is not convenient. Some people need to call taxis or rideshares which would increase their financial burden. With the development of the internet and the popularity of mobile phones, establishing patient-centered remote rehabilitation or home-based CR might be an effective way to alleviate this problem [35]. A study suggested that transporting staff and equipment to community settings might be a good way to overcome some of these barriers [36].

The cost of CR is a common barrier to attending CR, which is in accordance with a previous study in China [12]. According to the National Health Care Security Administration [37], China achieved 95% health coverage in 2020, and inpatients can be reimbursed for 70–80% of their medical expenses during hospitalization. However, outpatient participation in a CR program is not covered by the National Health Service. Therefore, some patients could not afford the CR program after hospital discharge. However, in most other countries, health insurance companies fully cover CR program costs [38, 39], and cost was not identified as a barrier in these countries. Therefore, it is better to include the CR program in the government insurance system to promote CR attendance. Additionally, considering the CR cost, more innovative and cost-effective possibilities should be explored.

Time conflict and work responsibilities were also identified as barriers in our study. Some elderly patients have to take on many family responsibilities, such as taking care of their grandchildren and doing housework for the families of their children’s generation. This is very normal in Chinese culture. For younger patients, time conflict refers to the need to work on workdays, and availability only on weekends. However, the CR center at the public hospital always closed on weekends and at night. Therefore, a flexible time schedule might be a way to improve CR attendance for elderly and working patients. Likewise, replacing some of the day classes with evening options could make it easier for some patients to attend.

In contrast to the findings in Liu’s study [13], bad weather and patients who did not know about CR were not identified as barriers in our study. This might be because our hospital is located in southern China with lovely weather in all four seasons. Moreover, the CR center in the hospital is designed to meet national standards. Inpatients will receive the standard phase 1 CR treatment and be referred to the phase 2 CR automatically if they are eligible. We have a specific educational session focused on introducing phase 2 CR before patient discharge. Therefore, most patients in our study had heard about CR during their hospitalization. However, in Liu’s study, 90% of participants had not even heard of CR. Therefore, inviting patients to take part early on during phase I CR and holding a specific educational session to introduce phase 2 CR might help create a space to motivate patients to participate in phase 2 CR. However, it is important to note that in our research, even though most patients had heard about CR, they did not participate in the phase 2 CR program. Just simply introducing CR without describing the program in detail is not always sufficient to motivate program participation.

The results from the qualitative phase confirmed most of the quantitative results and provided a deep explanation for the results. In addition, some new information also emerged in the qualitative phase. Parking difficulties were a barrier that emerged in the qualitative phase. This is in accordance with most previous research [40]. Many patients complained that it is quite difficult to find a parking space at the hospital. Parking space at the hospital is limited and not free. This might be a specific phenomenon to our local hospital, and it provides insight that hospitals should think about this issue ahead when establishing CR centers.

In addition, COVID-19 screening and testing were identified as barriers in our quantitative study phase. During the pandemic, our center did not stop CR program delivery; however, we did not provide any home-based programs due to limited resources and lack of standard home-based CR model. Patients needed to come to our on-site center 2–3 times a week, and every 72 hours, they needed to show a negative test result. Some of them complained that it is terrible to have to repeatedly do the nasal swab to show a negative result when they enter the center. A global cross-sectional study reported that during the pandemic, approximately 49% of CR programs had stopped CR delivery, and 25.7% of patients had to stop their exercise because they had no place to exercise [22]. Moreover, previous research reported that other barriers related to COVID-19 restriction arose, including concern about COVID-19, worry about the risk of infection and resistance to wearing a mask during exercise. Therefore, during the pandemic, some remote home-based programs might be explored to overcome some of these barriers. A current national cross-sectional study in UK reported that telephone was most commonly used to deliver cardiac rehabilitation, and some centers used sophisticated technology such as teleconferencing during the pandemic [41].

In the qualitative phase, two new themes emerged, namely, impressions of health care providers and hospital surroundings, psychological distress and personality. Some patients were referred by nurses whom they were not familiar with. Therefore, they were less likely to fully believe what the nurse told them. This reminds us that CR is a comprehensive program that requires the cooperation of a multidisciplinary team. Cardiologists play an important role in the referral process. The health care provider’s impression is also viewed as a barrier in this study. Some patients believe that nurses or physicians who guide CRs should be healthy and slim. They do not think a nurse who does not match their physical expectations can help them maintain a healthy weight. This is in accordance with some previous studies [42, 43], which reported that physician body mass index (BMI) is a potential barrier to obesity care. Physicians with a normal BMI were more likely to engage their obese patients in weight loss discussions than overweight/obese physicians. Physicians with a normal BMI had greater confidence in their ability to provide diet and exercise counseling to their obese patients. A high percentage of physicians with a normal BMI believed that overweight/obese patients would be less likely to trust weight loss advice from overweight/obese doctors. For some participants, their impression of the surrounding rehabilitation center also impacted their decision-making process.

Psychological distress and specific personality traits, such as hopelessness, pessimism, fractiousness, anger and disbelief of health care providers, were barriers to CR. Some participants expressed their suspicions about the program. They believed the purpose of the hospital was only to make money. This theme is in accordance with previous research and adds information on the relationship between psychological distress and poor CR attendance [44]. These findings, combined with the last theme (impressions of staff and the surroundings of CR centers), suggest that health care providers should recognize that psychological distress and personality might interfere with patients’ decision-making. Therefore, attending to the emotional context of prospective CR patients is quite important. In addition, some psychosocial assessments and interventions should be used to evaluate potential participants and build a good relationship between patients and health care providers. For example, some mindfulness-based practices are reported to be effective in improving the interpersonal relationship between patients and health care providers [45].

There are several implications for practice and research. First, this research identified the barriers to CR in the current Chaoshan region in China during the pandemic, which will provide evidence for intervention programs targeted at removing these barriers. Second, given the results of this study, which identified mainly logistical barriers, one possible solution is to improve the availability of home-based programs. Home-based programs are a safe and effective alternative for low- and moderate-risk patients. Hospital-based CR centers can explore suitable home-based CR, especially in this COVID-19 pandemic period.

## Limitations

There are several limitations in this study. First, the data were only from one university hospital. In the future, a multicenter study with a larger sample size is needed. Second, for the quantitative phase, CR barriers were assessed only on the day before patient discharge. Barriers might change after patients return to their usual life. Therefore, a continuous assessment after the patients’ discharge could provide more information. Third, generalizability. Whether the results are applicable to patients with CHD outside the city of Shantou or in other counties requires further study. Moreover, the results are only generalizable to inpatients who have been informed about CR and who are referred to a hospital center-based CR program. Patients with CHD who received a coronary artery bypass graft or who have different ethnic identities may have had different barriers than participants consenting to the study. Finally, the researcher’s personal lens might have impacted the interpretation of the qualitative themes. Therefore, to minimize these impacts, two researchers with interdisciplinary backgrounds read the transcripts independently and came to a consensus about the themes.

## Conclusion

This study involved collecting both quantitative and qualitative data and intentionally integrating the data to provide a better understanding of the barriers to hospital-based phase 2 CR programs among CHD patients in the Chinese context. Both the quantitative and qualitative phases confirmed that logical factors, such as distance, transportation, program cost, time conflict and responsibility, are the main barriers to participating in CR. The qualitative results provide a deep explanation of these barriers and reveal that impressions of CR teams, psychosocial distress and specific personality traits are barriers. Moreover, the COVID-19 testing restrictions were also a new barrier during this study period. Therefore, intervention programs aimed at promoting participation should focus on how to overcome these barriers. Some innovative methods, such as home-based CR, mobile health, and hybrid programs, might be effective in overcoming some of these barriers and improving the participation rate. In addition, psychological and social assessments are needed to evaluate the psychosocial status of patients, and some psychosocial intervention programs might be beneficial to overcome some of these barriers.

## Data Availability

The datasets generated and/or analyzed during the current study are not publicly available due to confidentiality but are available from the corresponding author on reasonable request.

## Abbreviations

CHD: Coronary Heart Disease
CVDs: Cardiovascular Diseases
CR: Cardiac Rehabilitation
PCI: Percutaneous Coronary Intervention
CABG: Coronary Artery Bypass Grafting
AMI: Acute Myocardial Infarction
STEMI: ST-elevation Myocardial Infarction
CRBS: Cardiac Rehabilitation Barriers Scale
COVID-19: Coronavirus disease 2019
CCU: Coronary care unit
